# Changes and Relationship of PAF and TNF in Rats with Myocardial Ischaemia and Reperfusion Injury

**DOI:** 10.1155/S0962935194000281

**Published:** 1994

**Authors:** Ya-Ling Han, Shao-Ha Li, Qin-Yue Zheng, Hong-Bin Wang, Guo-Yan Zhang, Zhong-Gui Wu, Si-Cong Chen

**Affiliations:** 1 Department of Cardiology Chang Zheng Hospital Shanghai China; 2 Department of Pathophysiology Second Military Medical Universit, Shanghai China; 3 Department of Pharmacology (School of Pharmacy) Second Military Medical University Shanghai China

## Abstract

In this study it is reported that: (1) the levels of blood platelet-activating factor and serum tumour necrosis factor significantly increased after coronary ligation and reperfusion, compared with sham-ligated controls, in an anaesthetized rat model; (2) compared with vehicle controls, pretreatment with the PAF antagonist BN 50739 (10 mg/kg, i.v.) produced significant decreases in infarct size (from 29.6 ± 4.0% to 22.4 ± 2.1%, p < 0.05 after 3 h ligation, and from 28.5 ± 9.5% to 10.5 ± 4.5%, p < 0.01 after 4 h reperfusion) and the level of serum TNF (from 10.4 ± 7.7 U/ml to 3.9 ± 4.8 U/ml, p < 0.05); and (3) a significan positive correlation was found between the level of blood PAF or serum TNF and infarct size. The present results indicate that PAF and TNF may be important mediators involved in myocardial ischaemia and reperfusion injury, and that PAF antagonists may exert a protective effect on ischaemic or reperfused myocardium by inhibiting the interaction of PAF and TNF.


Mediators of Inflammation 3, 205–209 (1994)

### Changes and relationship of PAF and TNF in rats with myocardial ischaemia and reperfusion injury

Ya-Ling Han,[1,CA] Shao-Ha Li,[2] Qin-Yue Zheng,[3] Hong-Bin Wang,[3] Guo-Yan Zhang,[1] Zhong-Gui Wu[1] and Si-Cong Chen[1]

[1]Department of Cardiology, Chang Zheng Hospital, Shanghai, China; Department of [2]Pathophysiology and [3]Pharmacology (School of Pharmacy), Second Military Medical University, Shanghai, China

[CA] Corresponding Author

**In this study it is reported that: (1) the levels of blood platelet-activating factor and serum tumour necrosis factor significantly increased after coronary ligation and reperfusion, compared with sham-ligated controls, in an anaesthetized rat model; (2) compared with vehicle controls, pretreatment with the PAF antagonist BN 50739 (10 mg/kg, i.v.) produced significant decreases in infarct size (from 29.6 ± 4.0% to 22.4 ± 2.1%, $p < 0.05$ after 3 h ligation, and from 28.5 ± 9.5% to 10.5 ± 4.5%, $p < 0.01$ after 4 h reperfusion) and the level of serum TNF (from 10.4 ± 7.7 U/ml to 3.9 ± 4.8 U/ml, $p < 0.05$); and (3) a significant positive correlation was found between the level of blood PAF or serum TNF and infarct size. The present results indicate that PAF and TNF may be important mediators involved in myocardial ischaemia and reperfusion injury, and that PAF antagonists may exert a protective effect on ischaemic or reperfused myocardium by inhibiting the interaction of PAF and TNF.**

**Key words:** Myocardial infarction, Platelet-activating factor, Reperfusion injury, Tumour necrosis factor

### Introduction

Evidence has recently been accumulated that platelet-activating factor (PAF) may contribute to myocardial ischaemia and reperfusion injury. It has been found that the myocardial infarct size, the incidence of arrhythmias and the mortality increase after exogenous administration of PAF to animals,[1] and that the level of serum tumour necrosis factor (TNF) markedly increases in patients with acute myocardial infarction.[2] However, the implication of increasing level of TNF and the relationship between PAF and TNF in myocardial ischaemia and reperfusion injury remain unclear. In order to explore the possible role of, and interaction between, PAF and TNF in myocardial ischaemia and reperfusion injury, the authors investigated: (1) the changes in blood PAF and serum TNF during myocardial ischaemia and reperfusion; (2) the correlation of PAF or TNF with myocardial infarct size; and (3) the possible protective effect of PAF antagonist BN 50739, in an anaesthetized open-chest coronary ligated and reperfused rat model.

### Materials and Methods

*Animals:* Sprague–Dawley male rats, weighing between 260 and 330 g, were obtained from SIPPR-BK Experimental Animal Cooperation (Shanghai, China). The rats were housed in the School of Pharmacy Animal Resources Center (Second Military Medical University) at 20 to 22°C and fed on standard rat chow and tap water *ad libitum*.

*Reagents:* Synthetic PAF, dimethylsulphoxide (DMSO) and 2,3,5-triphenyltetrazolium chloride (TTC) were obtained from Sigma Chemical Co. (St Louis, USA). BN 50739, a recently developed long-acting PAF receptor antagonist,[3] was kindly provided by Dr P. Braquet (Institut Henri Beaufour, Paris, France).

*Surgical procedures and experimental design:* The surgical technique described by Curtis[4] was used for coronary artery ligation and reperfusion with slight modifications. The rats were anaesthetized with amobarbital sodium at a dose of 45 mg/kg intraperitoneally, and then tracheotomized and ventilated (tidal volume, 1.5 to 2.0 ml; respiratory frequency, 70 per min) by using a Small Animal Respirator (Jiang-Wan Type 1, Second Military Medical University, Shanghai, China). A polyethylene catheter was introduced into the right carotid artery for measurement of blood pressure and for withdrawing blood samples. Another catheter was introduced into the left femoral vein for drug infusion. Standard lead of the electrocardiogram was recorded on an oscillographic recorder (Nihon Kohden, Tokyo, Japan). A thoracotomy was performed at the level of the fourth intercostal space. The pericardium was incised and the left main coronary artery was secured by a 5–0 silk suture around the artery approximately 2 mm from its origin. After completing the above surgical procedures, the rats were allowed to stabilize for 15 min. In this period, the rats that had arrhythmias or systolic pressure lower than 70 mmHg over 5 min

© 1994 Rapid Communications of Oxford Ltd were excluded from the experiment. The rats were then randomly divided into eight groups of which the details are summarized in Table 1. For Group 2, BN 50739, dissolved in DMSO and then diluted carefully during continuous stirring with normal saline, was injected intravenously 5 min prior to coronary ligation. Rats in Group 3 served as vehicle controls which accepted the same amount of DMSO and normal saline as Group 2. One min prior to reperfusion, BN 50739 was injected intravenously for Group 7, and the same amount of vehicle as Group 7 was injected for Group 8.

*Determination of myocardial infarct size:* The method described by Montrucchio[5] was used with slight modifications. At the end of the experiment, the heart was removed and immediately placed in 10% potassium chloride solution to induce rapid asystole. After the atria and blood vessels had been excised, the heart was cut into four sections from base to apex, each approximately 2 mm thick. The sections were then washed with cold saline and stained with freshly prepared 1% TTC in 0.2 M phosphate buffer (pH 7.4) at 37°C for 15 min. They were then washed in water, weighed and placed in 40% formalin solution for fixation. The sections were photographed using a Nikon F4 camera (Tokyo, Japan) within 1 week, and their photographs were used to measure the areas of infarcted myocardium with a computerized image analysis system (Automation Institute of China, Peking) according to the weight of each section.

*Determination of blood PAF:* The method for the extraction, purification and measurement of PAF in whole blood[6] was used. A blood sample (1.5 ml) drawn from the carotid artery at the end of experiment was immediately placed into 4 ml of methanol at 4°C for 2 h. The precipitated proteins were removed by vortex shaking for 30 s and then centrifugation for 10 min at 3 000 rpm, and 1.5 ml each of chloroform and water were added to the supernatant to effect phase separation. After a second vortex shaking and centrifugation, the lower, chloroform phase was dried completely under nitrogen and kept at –40°C until measurement. In all samples, PAF was purified by thin layer chromatography (chloroform: methanol: water, 65:35:6, v/v). Synthetic PAF was used as a standard and the treated samples were subjected to a biological measurement of PAF by aggregation of washed rabbit platelets.[6]

*Assay of serum TNF:* Blood samples (1 ml) collected at the end of the experiment were kept at 4°C for 2 h and then centrifuged at 3 000 rpm for 15 min. Serum was then separated and kept at –40°C until assayed. The activity of serum TNF was measured by its cytotoxic activity on murin L-929 cells in the presence of actinomycin D.[7] Briefly, L-929 cells were seeded at a density of $5 \times 10^4$ cells per well in 96-well plastic tissue culture plates (Nunclon Co., Denmark). The plates were incubated with 5% carbon dioxide at 37°C in an incubator (Heraeus Co., Hanau 1, Germany) for 12 h to produce a single cell line. One hundred µl of the 1-fold diluted serum to be assayed with actinomycin D (1 ng/ml of the final concentration) were added to the plates, and then incubated for 18 h. After the supernatant was removed the cells were stained with 0.5% crystal violet for 20 min, the plates were rinsed and dried. The absorbance of the extract was measured at 570 nm using an enzyme-linked immunoassay autoreader (DG-3022, East China Electron-Tube Factory, Xian). A concentration of 1 U/ml of TNF represents the reciprocal of the TNF dilution which causes 50% cytotoxicity under the assay conditions.

*Statistical analysis:* Data are presented as the mean ± 1 S.D. Intergroup comparisons for the myocardial infarct size and the levels of PAF and TNF were assessed, as appropriate, by a two-tailed analysis of variance or Student's *t*-test. A Fisher's analysis was used to assess the difference of percentage of animals with detectable PAF and TNF. A linear correlation analysis was used to assess the relationship between the infarct size and the level of PAF and TNF.

**Table 1.** Experimental groups of rats subjected to coronary artery ligation and reperfusion

| Group | *n* | Procedure | Treatment | Measurement |
|---|---|---|---|---|
| 1 | 9 | 3 h sham-ligation | None | PAF and TNF |
| 2 | 9 | 3 h ligation | BN 50739 10 mg/kg | IS, TNF and PAF |
| 3 | 10 | 3 h ligation | Vehicle | IS, TNF and PAF |
| 4 | 11 | 4 h ligation | Vehicle | PAF and TNF |
| 5 | 10 | 2 h ligation | Vehicle | PAF and TNF |
| 6 | 10 | 1 h ligation | Vehicle | PAF and TNF |
| 7 | 9 | 40 min ligation followed by 4 h reperfusion | BN 50739 10 mg/kg | IS, TNF and PAF |
| 8 | 10 | | Vehicle | IS, TNF and PAF |

IS = infarct size.

**Table 2.** Changes of blood PAF and TNF

| | Sham-ligated group | Coronary artery | | ligated group | | Reperfused group |
|---|---|---|---|---|---|---|
| | | 1 h | 2 h | 3 h | 4 h | 4 h |
| PAF (pg/ml) | 36 ± 59 | 348 ± 112[b] | 689 ± 199[b] | 625 ± 183[b] (593 ± 102) | 571 ± 201[b] | 1421 ± 454[b] (1399 ± 219) |
| TNF (U/ml) | 1.5 ± 1.6 | 4.2 ± 2.5[a] | 4.4 ± 2.1[b] | 10.4 ± 7.7[b] (3.9 ± 4.8)[c] | 8.3 ± 4.1[b] | 16.2 ± 5.7[b] (6.6 ± 4.1)[d] |

$n = 9$ for all the groups. The data in brackets were obtained from groups treated with BN 50739, the rest from vehicle controls. [a]$p < 0.05$, [b]$p < 0.01$, sham-ligated compared with ligated and reperfused vehicle controls. [c]$p < 0.05$, [d]$p < 0.01$, BN 50739 pretreated compared with vehicle controls.

### Results

*The changes of blood PAF and serum TNF:* The changes of blood PAF and serum TNF are summarized in Table 2. In vehicle controls, the level of blood PAF significantly increased after 1 h myocardial ischaemia, reached its peak level which was 19 times that of sham-ligated controls after 2 h myocardial ischaemia, and slightly decreased after 3 and 4 h ischaemia but still remained significantly higher compared with sham-ligated controls. The level of blood PAF after 4 h reperfusion increased further compared with those of ischaemic groups, which was about 40 times that of sham-ligated controls. The level of blood PAF in groups pretreated with BN 50739 before ischaemia and reperfusion was not significantly changed compared with vehicle controls subjected to ischaemia and reperfusion for the same time period. A minimal amount of PAF was detected in the blood obtained from three of nine rats (33.3%) in the sham-ligated group. PAF was detected in all animals that underwent coronary artery ligation or reperfusion. The differences in the percentage of animals with detectable blood PAF in sham-ligated and the coronary ligated or reperfused groups were significant ($p < 0.01$).

In the vehicle controls, the level of serum TNF increased significantly at 1 h myocardial ischaemia, reached a peak level that was seven times that of sham-ligated control after 3 h ischaemia, and decreased after 4 h ischaemia but remained significantly higher compared with sham-ligated controls. The level of serum TNF in the coronary reperfusion group without BN 50739 pretreatment exceeded those in the ischaemic groups, which was eleven times that of sham-ligated controls. But in the groups pretreated with BN 50739 before ischaemia and reperfusion the TNF level decreased significantly compared with vehicle controls after the same time period of ischaemia and reperfusion. Serum TNF was detected in five of nine (55.6%) rats in the sham-ligated group, but was detected in all but two rats subjected to coronary artery ligation or reperfusion; in two of the nine rats pretreated with BN 50739 and subjected to 3 h coronary ligation TNF was not detected. The differences in the percentage of animals showing detectable serum TNF between sham-ligated and the coronary artery ligated groups without BN 50739 pretreatment or both reperfused groups were significant ($p < 0.05$). However, the difference between the sham-ligated group and the 3 h ligated group pretreated with BN 50739 was not significant.

*Effect of BN 50739 on infarct size:* As shown in Table 3, the infarct size significantly decreased in both the 3 h ischaemia group and the 4 h reperfusion group pretreated with BN 50739 compared with the respective vehicle controls. The weights of left and right ventricles in all groups were not significantly different.

Linear correlation analysis showed that the correlation coefficients between blood PAF level and infarct size for Groups 2 and 3 ($n = 18$), and Groups 7 and 8 ($n = 18$) were 0.502 ($p < 0.05$) and 0.614 ($p < 0.01$), respectively. Between serum TNF and infarct size correlation coefficients for Groups 2 and 3 ($n = 18$), and Groups 7 and 8 ($n = 18$) were 0.479 ($p < 0.05$) and 0.608 ($p < 0.01$), respectively. Correlation coefficients between the levels of PAF and TNF were 0.469 ($p < 0.05$) for Groups 2 and 3 ($n = 18$), and 0.698 ($p < 0.01$) for Groups 7 and 8 ($n = 18$).

### Discussion

In this study it was found that the level of blood PAF in the rats with coronary artery ligation from 1 to 4 h increased significantly, and increased further in the rats subjected to 40 min coronary ligation followed by a 4 h reperfusion, indicating that synthesis and release of PAF may increase during acute

**Table 3.** Effects of BN 50739 on infarct size in rats subjected to coronary artery ligation and reperfusion

| Group | *n* | Treatment | WV (mg) | IS (mg) | IS/WV (%) |
|---|---|---|---|---|---|
| 2 | 9 | BN 50739 | 902 ± 170 | 220 ± 4[a] | 24.4 ± 2.1[a] |
| 3 | 8 | Vehicle | 872 ± 169 | 258 ± 7 | 29.6 ± 4.0 |
| 7 | 9 | BN 50739 | 906 ± 146 | 95 ± 7[b] | 10.5 ± 4.5[b] |
| 8 | 9 | Vehicle | 882 ± 144 | 251 ± 14 | 28.5 ± 9.5 |

IS = infarct size; WV = weight of left and right ventricles. [a]$p < 0.05$, [b]$p < 0.01$, compared with vehicle groups.

myocardial ischaemia and reperfusion. A strong positive correlation existed between infarct size and blood PAF level. BN 50739, a selective PAF receptor antagonist, significantly reduced the infarct size in the rats with 3 h coronary artery ligation and 4 h reperfusion. This suggests that PAF may be an important mediator involved in myocardial ischaemia and reperfusion injury. There is evidence that platelets, leukocytes, endothelial cells and cardiomyocytes subjected to the stimuli of ischaemia and hypoxia are very active in generating PAF.[8,9] The mechanism by which PAF aggravates ischaemia and reperfusion injury to the myocardium has not been wholly clarified. It was reported that PAF, besides inducing platelets located in the ischaemic myocardial region to release vasoactive and thrombogenic substances (thromboxane $A_2$, etc.),[1] could increase the number of polymorphonuclear leukocytes by its chemotaxic action in the ischaemic region, activate these cells to release oxygen free radicals and leukotrienes,[1,10] and induce the dysfunction of endothelial cells (especially the coagulative dysfunction).[11] These effects of PAF may contribute to the myocardial damage during ischaemia and reperfusion.

At a very low concentration ($10^{-16}$ M) PAF can release TNF from cultured monocytes/macrophages,[12] while cultured endothelial cells and neutrophils generate PAF and other cytokines when stimulated by TNF.[1] TNF enhances the procoagulant activity of endothelial cells, induces shock and tissue injury, and increases vascular permeability and lethal effects of PAF in animals.[13] Therefore, TNF might aggravate ischaemic injury and extend myocardial infarct size. In view of these facts, TNF might have a close functional similarity to PAF in myocardial ischaemia injury. The authors found that the percentage of animals exhibiting an increase in blood concentration of PAF and TNF increases simultaneously during myocardial ischaemia and reperfusion, and that infarct size was significantly correlated with both PAF and TNF levels. This suggests that PAF and TNF generated in a mutually stimulated way and had synergic harmful effects on ischaemic and reperfused myocardium. We also found that the maximal level of TNF appeared 1 h later than that of PAF, indicating that PAF might be released first in myocardial ischaemia and then stimulate the generation of TNF, and thus PAF might have a potential central role in the interaction between PAF and TNF.

Interestingly, the levels of PAF and TNF after 4 h reperfusion were higher than the maximal levels in rats with myocardial ischaemia. One possible explanation for the phenomenon is that larger amounts of PAF and TNF, generated and accumulated in the ischaemic region, were released into blood when reperfusion started. Another possibility is that PAF and TNF were synthesized and released during the reperfusion period. Pathophysiologically, reperfusion injury may partially differ from ischaemic injury in that a large number of inflammatory cells (mainly polymorphonuclear leukocytes) infiltrate and are trapped in the ischaemic area and then adhere increasingly to endothelial cells during reperfusion, resulting in a 'no reflow region'. The correlations between infarct size and level of PAF or TNF in the rats undergoing reperfusion were more significant than in the rats undergoing ischaemia in the present study, indicating that PAF and TNF may contribute more to injury resulting during myocardial reperfusion than that during ischaemia. The effect of BN 50739 in reducing infarct size observed in the present study indicates that BN 50739 may behave as a cellular protective agent and prevent more cardiomyocytes from irreversible ischaemia and reperfusion injury. BN 50739 may exert its effect by closely binding itself to the PAF receptor in the target organs, since the level of blood PAF in the rats with ischaemia or reperfusion pretreated with BN 50739 did not differ from that in the vehicle controls, but the reduction in the infarct size was remarkable. It is worth emphasizing that the level of serum TNF in the rats with ischaemia and reperfusion pretreated with BN 50739 decreased significantly when compared with vehicle controls in this study, supporting further that a positive feedback of interaction between PAF and TNF existed and that a part of the biological activity of PAF was mediated via TNF.

In conclusion, the present results suggest that both PAF and TNF are important mediators involved in myocardial ischaemia and reperfusion injury. They prime each other's generation and release and exert synergic harmful effects on the ischaemic and reperfused myocardium. The PAF receptor antagonist, BN 50739, may inhibit the positive feedback of PAF in releasing TNF, protect cardiomyocytes against the injury, and thus decrease infarct size when administered before ischaemia and reperfusion. Further studies may be needed to define whether the combination of PAF antagonist with TNF antibodies could produce more benefit in the prevention and treatment of myocardial ischaemia and reperfusion injury.

### References

1. Koltai M, Hosford D, Guinot P, Esanu A, Braquet P. Platelet-activating factor (PAF), a review of its effects, antagonists and possible future clinical implications (Part 1). *Drugs* 1991; **42**: 9–29.
2. Maury CPJ, Teppo AM. Circulating tumor necrosis factor—a (Cachetin) in myocardial infarction. *J Intern Med* 1989; **225**: 333–336.
3. Yue TL, Rabinovici R, Farhat M, Feuerstein G. Inhibitory effect of new PAF antagonists on PAF induced rabbit platelet aggregation *in vitro* and *ex vivo*. *J Lip Med* 1991; **3**: 13–26.
4. Curtic MJ, Macleod BA, Walk MJA. Models for the study of arrhythmias in myocardial ischemia and infarction: the use of the rats. *J Mol Cell Cardiol* 1987; **19**: 399–419.
5. Montrucchio G, Alloatti G, Mariano F, *et al.* Role of platelet-activating factor in the reperfusion injury of rabbit ischemic heart. *Am J Pathology* 1990; **137**: 71–83.
6. Li SH, Zhang HH, Chen SF, Ding ZQ, Fei X, Wu ZL. Platelet-activating factor mediates endotoxin-induced canine lung injury and its mechanisms. *J Med Coll PLA*

1992; **4**: 374–381.

7. Hirai M, Okamura N, Terano Y, Tsujimoto M, Nakazato H. Production and characterization of monoclonal antibodies to human tumor necrosis factor. *J Immunological Methods* 1987; **96**: 57–62.
8. Joseph R, Welch KMA. Granulocytes, platelet-activating factor, and myocardial injury. *Circulation* 1989; **79**: 1401.
9. Janero D, Burghardt C. Production and release of platelet-activating factor by the injured heart-muscle cell (cardiomyocyte). *Res Commun Chem Path Pharm* 1990; **67**: 201–215.
10. Ma XL, Weyrich AS, Krantz S, Lefer AM. Mechanism of the cardioprotective actions of WEB-2170, bepafant, a platelet activating factor antagonist, in myocardial ischemia and reperfusion. *J Pharm Exp Ther* 1992; **260**: 1229–1236.
11. Braquet P, Hosford D, Braquet M, Bourgain RH, Bussoline F. Role of cytokines and platelet-activating factor in microvascular immune injury. *Int Arch Allergy Appl Immunol* 1989; **88**: 88–100.
12. Braquet P, Braquet M, Bourgain RH, Bussolino F, Hosford D. PAF/cytokine autogenerated feedback networks in microvascular immune injury: consequences in shock, ischemia and graft rejection. *J Lip Med* 1989; **1**: 75–112.
13. Sirois MG, Plante GE, Braquet P, Sirois P. Tumor necrosis factor primed the effects of platelet-activating factor on rat vascular permeability. *J Lip Med* 1990; **2**: S109–S117.

**Received 21 January 1994; accepted in revised form 8 March 1994**
